# Novel Interventional Management of Hepatic Hydatid Cyst with Nanosecond Pulses on Experimental Mouse Model

**DOI:** 10.1038/s41598-017-04873-5

**Published:** 2017-07-03

**Authors:** Xinhua Chen, Ruiqing Zhang, Tuerganaili Aji, Yingmei Shao, Yonggang Chen, Hao Wen

**Affiliations:** 1 Department of Hepatobiliary and Pancreatic Surgery, The First Affiliated Hospital, Zhejiang University, Hangzhou Zhejiang, 310003 China; 2 Collaborative Innovation Center for Diagnosis and Treatment of Infectious Diseases, The First Affiliated Hospital, Zhejiang University, Hangzhou Zhejiang, 310003 China; 3 Key Laboratory of Combined Multi-organ Transplantation, Ministry of Public Health, The First Affiliated Hospital, Zhejiang University, Hangzhou Zhejiang, 310003 China; 4 Hepatobiliary & Hydatid Department, Digestive and Vascular Surgery Centre, Xinjiang Key Laboratory of Echinococcosis, The First Affiliated Hospital of Xinjiang Medical University, Urumqi, Xinjiang 830011 China; 5 Xinjiang Nanosecond Pulsed Power Technology Institute, Urumqi, Xinjiang 830000 China

## Abstract

The nanosecond pulsed electric field (nsPEF) is investigated as an alternative plan for benign hepatic hydatid cyst. Altogether 72 C57B6 mice were included. Normal group (n = 12) had no parasite injection and the other 60 mice were used to induce hydatid cyst in liver by injecting protoscolices in portal vein. The liver hydatid cysts were exposed to nsPEF with different doses and then follow up. The standard surgery was performed as positive control. The hydatid cyst growth was monitored by ultrasound; the morphology was checked by gross anatomy and pathology was tested by H&E stain. In nsPEF-treated groups no hepatic failure nor bleeding were observed. As a comparison, in the surgery group, high post-treatment complications occurred (50%). Significant parasite growth inhibition was seen in high nsPEF dose group as compared with control group (P < 0.05). Pathological analysis confirmed destruction of hydatid cyst with sharp demarcation defined by the electrodes. Laboratory analysis showed nsPEF stimulated a time-dependent infection and recoverable liver function. The traumatic reactions defined by white blood count was significant lower than surgery groups (P < 0.05).Preliminary studies demonstrate nsPEF ablation can be applied on hepatic hydatid by inhibiting parasite growth, destructing the cyst and stimulating infections.

## Introduction

Human echinococcosis is a serious zoonotic disease worldwide distributed that jeopardizing human health and economic development. It is caused by the tapeworm of echinococcosis larvae in humans and sheep. It has been found in every continent except Antarctica and highly epidemic in the areas of animal husbandry development e.g. rural China, Russia, continental Europe and North America^1^. In China, it affects 5% of the national population. About 100 million livestock animals are subjected to disease infection; among them more than 70% are sheep^2^. Cystic echinococcosis has been classified as one of the major parasitic diseases to be controlled urgently by 2020 by dog management, slaughterhouse hygiene control, health education and human patient treatment^3^.

Currently the effective treatments against human cystic echinococcosis include chemotherapy and surgery. Due to the low absorption, the chemotherapy can only be used as an adjuvant to surgical treatment. Radical surgery is regarded as the most effective treatment but surgery causes severe complications such as biliary vessel infection, residual cyst abscess, interoperation planting and contamination^4^. The WHO guideline suggest stage-specific treatment, e.g. uncomplicated cysts of the liver should be treated by non-surgical options such as percutaneous drainage accompanied with chemotherapy^5^.

**Figure 1:**
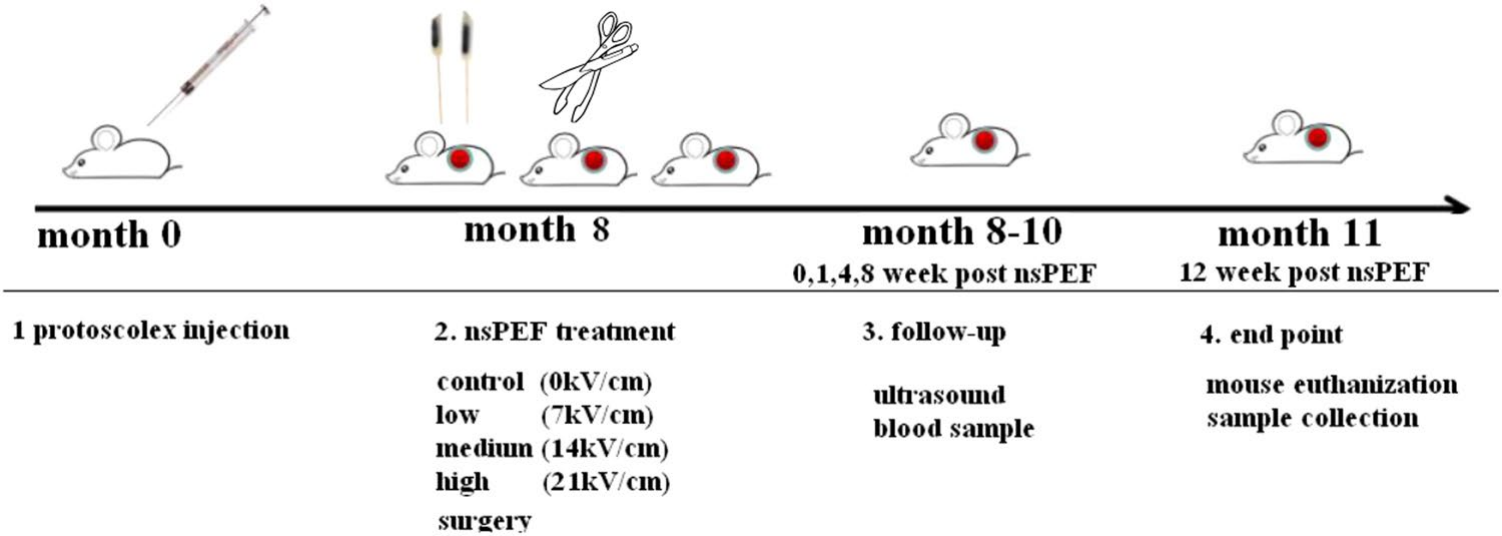
Experiment time course and interventions on animals. It took total of 12 months to complete the animal experiment which include two stages: (1) The protoscolex injection in portal vein. (2) nsPEF treatment. (3) post-treatment follow up and (4) end point to euthanize the animal and collect samples.

Percutaneous treatment PAIR (puncture aspiration injection re-aspiration) was introduced decades ago with fewer complications and less cost. Percutaneous techniques are particularly suitable for unilocular cysts, with or without detached endocysts^6^. Single cystic echinococcosis has big parasite cyst with high cyst fluid tension and active protoscolices, showing the highest risk of post-surgery recurrence. So in this study the single cystic echinococcosis was set up in the mouse liver as the experimental model to ablate the orthotopic cystic echinococcosis by nsPEF treatment *in vivo* (Fig. 1).

**Figure 2:**
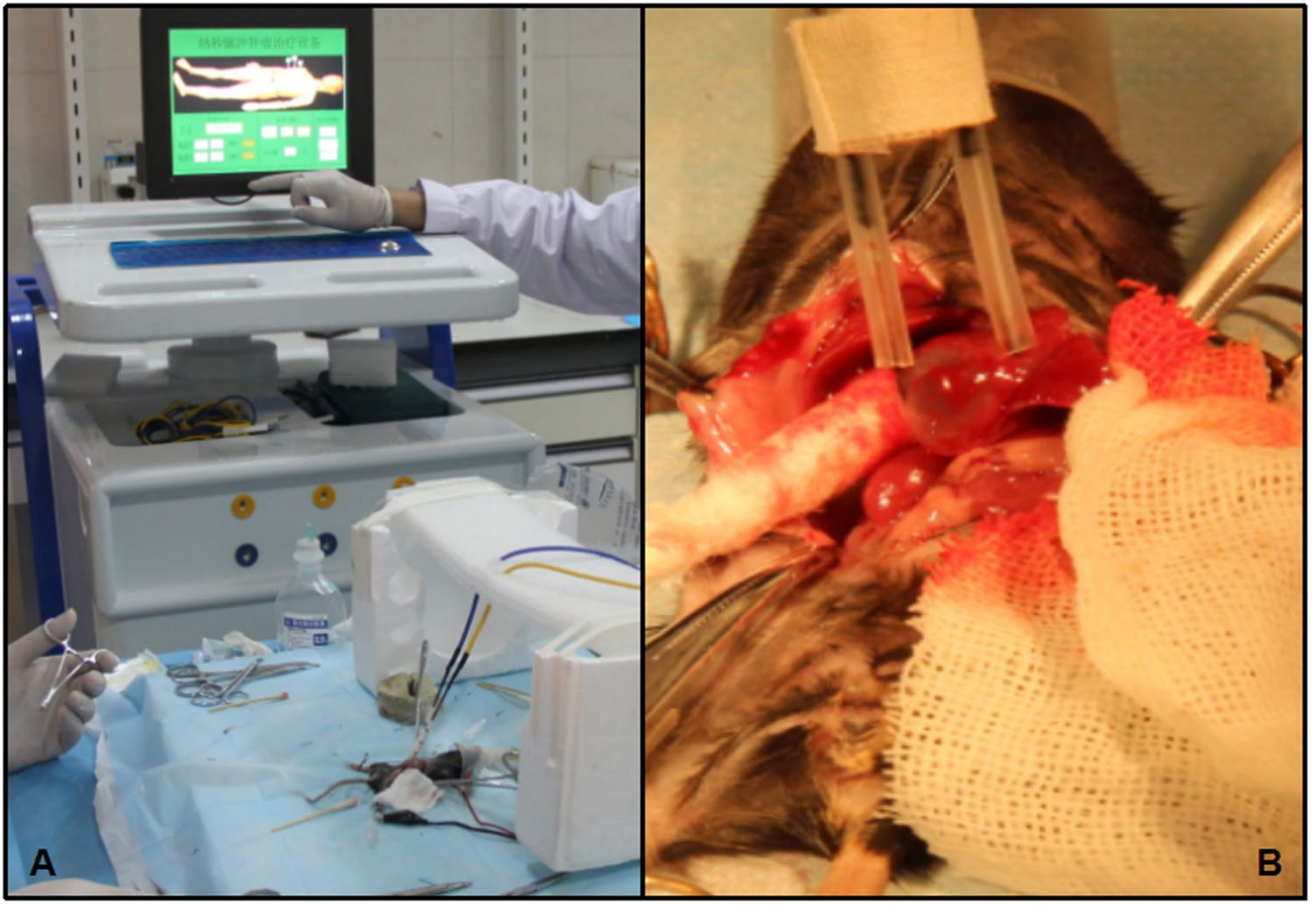
The nsPEF instrument and ablation electrodes. The nsPEF generator (Fig. 2A) and electrode (Fig. 2B) were applied on C57/B6 mice with parasite cyst in liver.

Nanosecond pulsed electric field (nsPEF) is a novel tumor ablation technology. By using the minimally invasive electrodes, high pulsed power is delivered with ultra-short electric pulses. The external electric field changes cell membrane potential, disrupting cell membrane structure and function, leading various anti-tumor effects. It has been proved effective in malignant tumors such as melanoma, skin tumor, and hepatocellular carcinoma^7^. In this study, for the first time, the nanosecond pulse with different voltages (Fig. 2) was applied to investigate the biological effect, feasibility and safety of using nsPEF ablating benign cyst lesion, *echinococcus granulosus* in liver.

## Results

### The growth of *echinococcus granulosus* in mouse liver after different dose of nsPEF treatment

**Figure 3:**
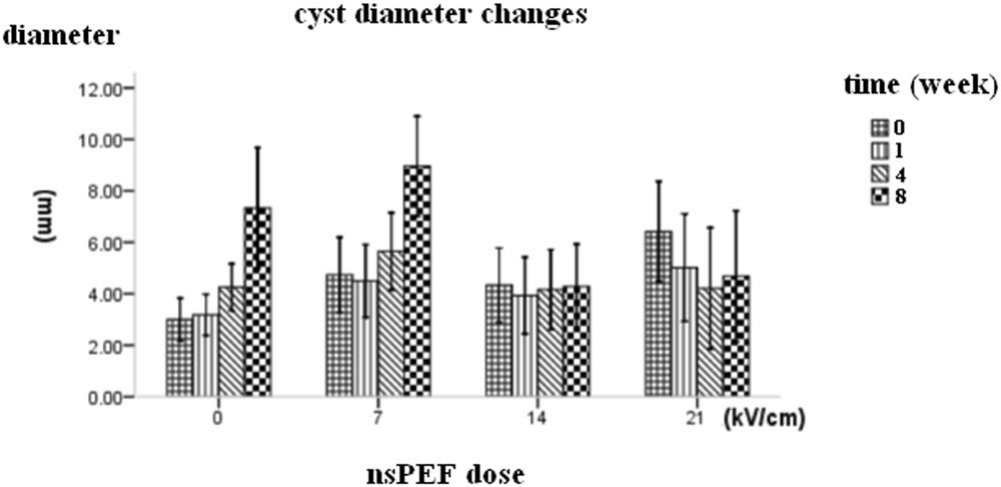
The parasite cyst growth over time after different nsPEF dosages. The diameter of mouse hepatic cystic echinococcosis was measured by ultrasound before and after nsPEF treatment. The average diameter on the 1^st^, 4^th^ and 8^th^ week post nsPEF treatment in different dose groups were recorded and compared.

The diameter of mouse hepatic cystic echinococcosis was measured by ultrasound. In the high electric field strength group (21 kV/cm group), the average diameter on the 1^st^, 4^th^ and 8^th^ week post nsPEF treatment was 5.02 ± 2.50 mm, 4.21 ± 2.82 mm and 4.68 ± 3.04 mm respectively. In the medium electric field strength group (14 kV/cm group), the average cyst diameter on the 1^st^, 4^th^ and 8^th^ week post nsPEF treatment was 3.93 ± 1.78 mm, 4.16 ± 1.86 mm and 4.28 ± 1.97 mm; In the low electric field strength group (7 kV/cm group), the average diameter on the 1^st^, 4^th^ and 8^th^ week post nsPEF treatment was 4.50 ± 1.34 mm, 5.56 ± 1.21 mm and 8.96 ± 1.56 mm, respectively; the control group on the 1^st^, 4^th^ and 8^th^ week was 3.18 ± 0.96 mm, 4.26 ± 1.08 mm, and 7.33 ± 2.81 mm, respectively (Fig. 3).

The average diameter increase of the high dose nsPEF group (21 kV/cm) increased −1.4 mm, −1.2 mm, −1.7 mm on the 1^st^, 4^th^ and 8^th^ week after nsPEF treatment; The average diameter increase of medium dose of nsPEF treatment group (14 kV/cm) was−0.4 mm, 0.4 mm, −0.1 mm on the 1^st^, 4^th^ and 8^th^ week post nsPEF treatment respectively. The mean diameter increase of the of low dose of nsPEF treatment group (7 kV/cm) was 0.2 mm, 1.3 mm, 4.3 mm on the 1^st^, 4^th^ and 8^th^ week after nsPEF treatment, respectively. The mean diameter increase of the control group were 0.2 mm, 1.3 mm, 4.3 mm on the 1^st^, 4^th^ and 8^th^ week after nsPEF treatment, respectively.

**Figure 4:**
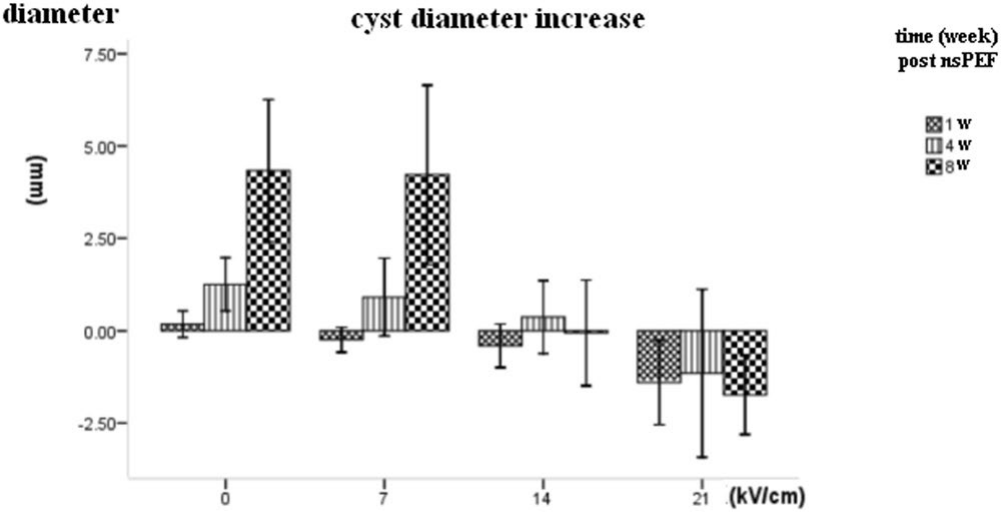
The diameter increase of mouse hepatic cystic echinococcosis lesion over time after different nsPEF dosages. To better reflect the growth tendency, the cyst diameter increase on the 1st, 4th and 8th week after nsPEF treatment were measured and calculated.

Figures 3 and 4 showed the cyst size had a significant difference between the high dose nsPEF group (21 kV/cm) and the control group (P < 0.05). The medium nsPEF dose (14 kV/cm) can only decrease the parasite cyst growth significantly on the 8^th^ week compared with the control group at the same time (P < 0.001). The diameter of *echinococcus granulosus* in the 7 kV/cm pulsed group showed no significant size difference vs control group (Fig. 3 and Fig. 4).

### Gross anatomical changes of echinococcus granulosus in mouse liver before and after nsPEF treatment

**Figure 5:**
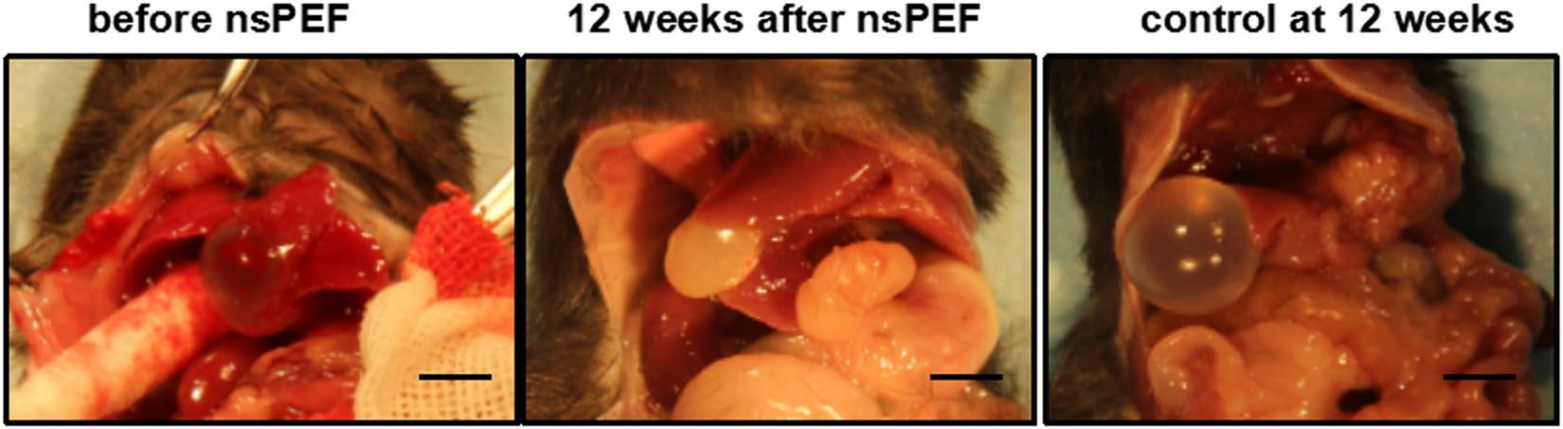
The gross anatomy changes of mouse cystic echinococcosis lesion. During the open check, the real appearance of mouse cystic echinococcosis lesion before and after nsPEF treatment were imaged for comparison. The scale bar is 1 cm.

Because there was an elastic capsule which can well define the parasite lesion in the liver parenchyma, the hydatid cyst’s outer appearance was accessible and consistent in ultrasound image series. Sonographic measurement of its maximum length was recorded. A typical parasite cyst was imaged as a round homogeneous lesion with a delicate thin wall. After 12 weeks post nsPEF treatment, the cyst fluid volume reduced and the capsules became wrinkled; the cystic wall became irregular and the cyst content changed into debris; the thin cyst wall became thickened; the transparency disappeared, the cyst fluid changed from liquid into solid; The control mice had the expanded echinococcus cyst with smooth and transparent, thin wall with elasticity and fluid tension (Fig. 5).

### The dynamic morphological and pathological changes of *Echinococcus granulosus* in mice after nsPEF treatment

**Figure 6:**
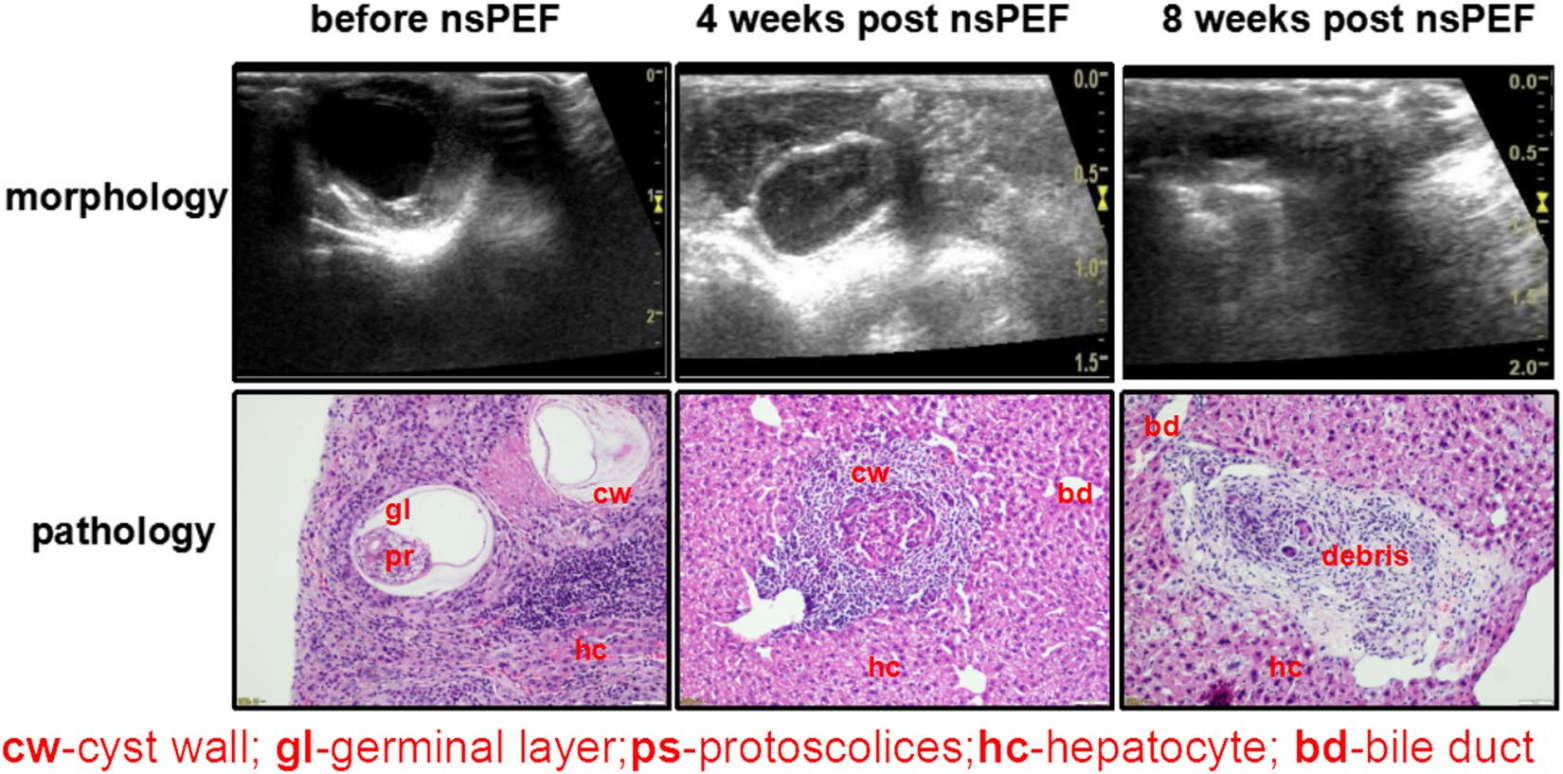
The pathological biopsy and ultrasound follow up after nsPEF. The pathological biopsy and ultrasound follow after nsPEF. The scale bar in ultrasound was present as indicated. The scale bar in pathological slides was 50 μm. Before treatment, the hydatid cysts showed active germinal layer and protoscolices. The enlarged parasite cysts pressed the hepacytes and congested the portal veins. Four weeks post nsPEF ablation, the ablated hydatid cyst showed focal degeneration without any germinal layers or protoscolices. Eight weeks post nsPEF ablation, mouse liver showed protective immune reactions, such as lymph cell infiltration and fibrosis capsules for the final clean up of the residual debris.

Without nsPEF, the parasite cyst present as a multiple layer-structure with a clear thin outline without any cracks (Fig. 6). At the 4^th^ week post nsPEF, the parasite cyst wall became thickened. Around the outer fibrous tissue of echinococcus, the inflammatory reaction zone formed. At the 8th week post nsPEF treatment, the *echinococcus granulosus* was disordered, the continuity of the cuticle was destroyed, the lamellar structure was disrupted and lamellar layer was broken.

The pathological changes corresponded to the changes of ultrasound image characters. Before nsPEF, the acellular laminated layer and thin cellular, germinal layer with brood capsules and protoscolices can often be identified by H&E staining. After nsPEF, the boundary of the ablation area was clear. Around the ablation area, an inflammatory reaction zone formed. After 4 week post nsPEF treatment, the infiltration of inflammatory cells around the ablation area increased; After 8 weeks post nsPEF, the infiltration of inflammatory cells further aggravated and infiltrated into the debris (Fig. 6).

### The laboratory follow -up of infection and liver function post nsPEF treatment

**Figure 7:**
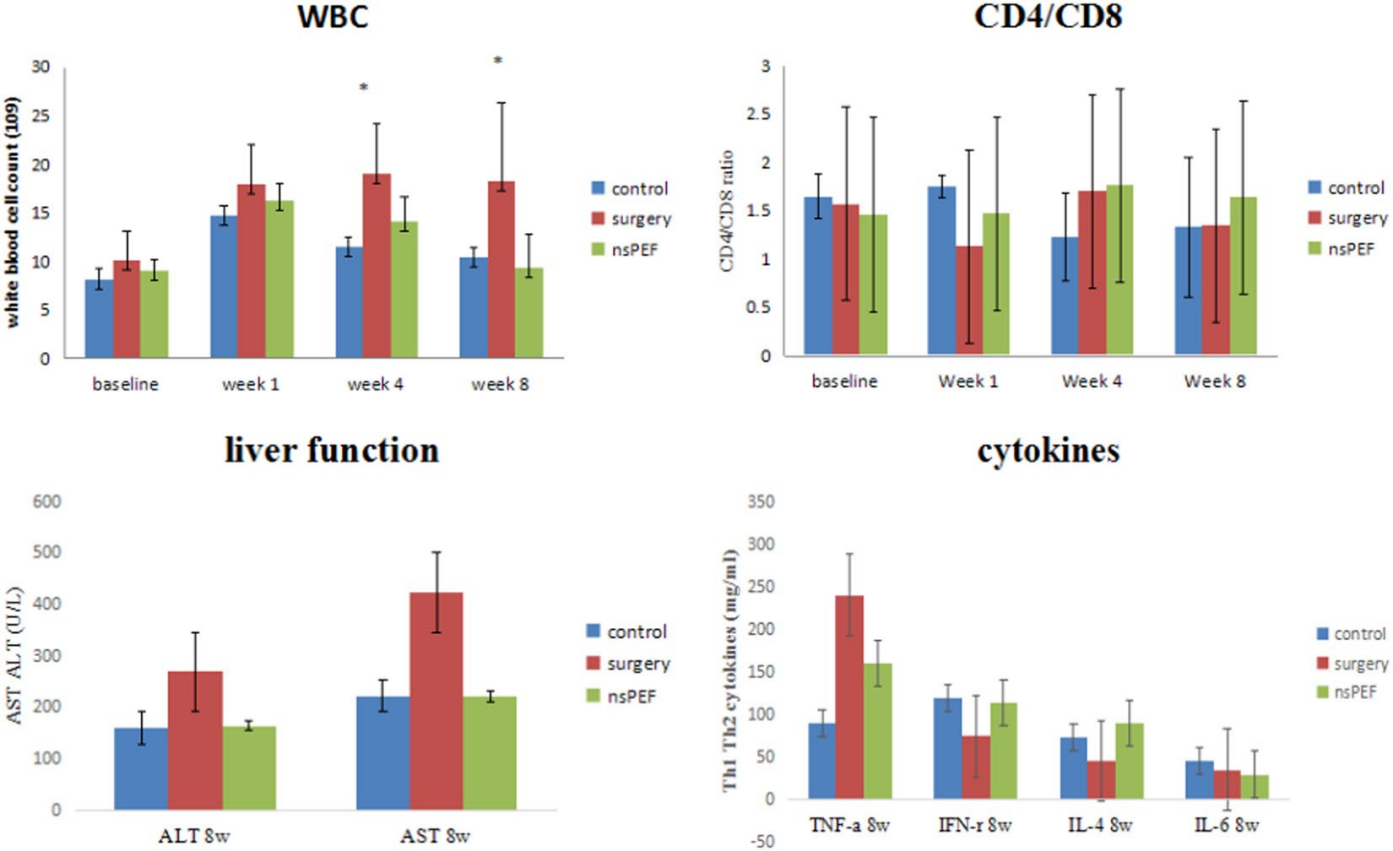
The laboratory follow -up of infection and liver function post nsPEF treatment. Mouse serum were collected at different time points after infection as indicated, Quantitative results of WBC before nsPEF, 1 week, 4 weeks and 8 weeks post nsPEF were shown *P < 0.05, between the indicated groups. Values were expressed as the mean ± SEM from 3 independent measurements.

Because the hydatid cysts in our study is the CE1 type cysts, the complete cyst wall protects the inner parasite antigens from the host’s immune system, serological test on immunological antibodies against specific parasite antigens such as protoscolex and cyst fluid were negative. So the nonspecific hematological white blood cells were tested to reflect the general inflammation. As shown in Fig. 7 at the 1^st^ week post nsPEF, WBC increased significantly in surgery group, control group (sham) and nsPEF group vs baseline(P < 0.05). While over time, from 4 weeks to 8 weeks post nsPEF, WBC declined in nsPEF and control group but remained high in surgery group. cytokines were follow up to monitor possible post treatment cytokine storm Th1 and Th2 type cytokines were tested and showed no significant changes, indicating the current nsPEF dosage did not break immune tolerance formed in chronic parasite infection (Fig. 7).

After 12 weeks post nsPEF, liver function marked by ALT and AST showed significant increase both in control and nsPEF group compared with the healthy mice (P < 0.05). But there was no difference between control group and nsPEF group, indicating that the liver function disorder came from as the comprehensive effect of parasite, electrode puncture and nsPEF ablation, indicating the damage of nsPEF caused to the liver is not as significant as surgery group. The non-thermal ablation did not cause massive necrosis and the residual liver lobes reacted with clean regeneration without cytokine storms. The pathological characters were in accord with laboratory exams. The trauma caused by surgery result in inflammatory situations. Marked by leukocyte surge (Fig. 7).

### Post treatment complications

**Figure 8:**
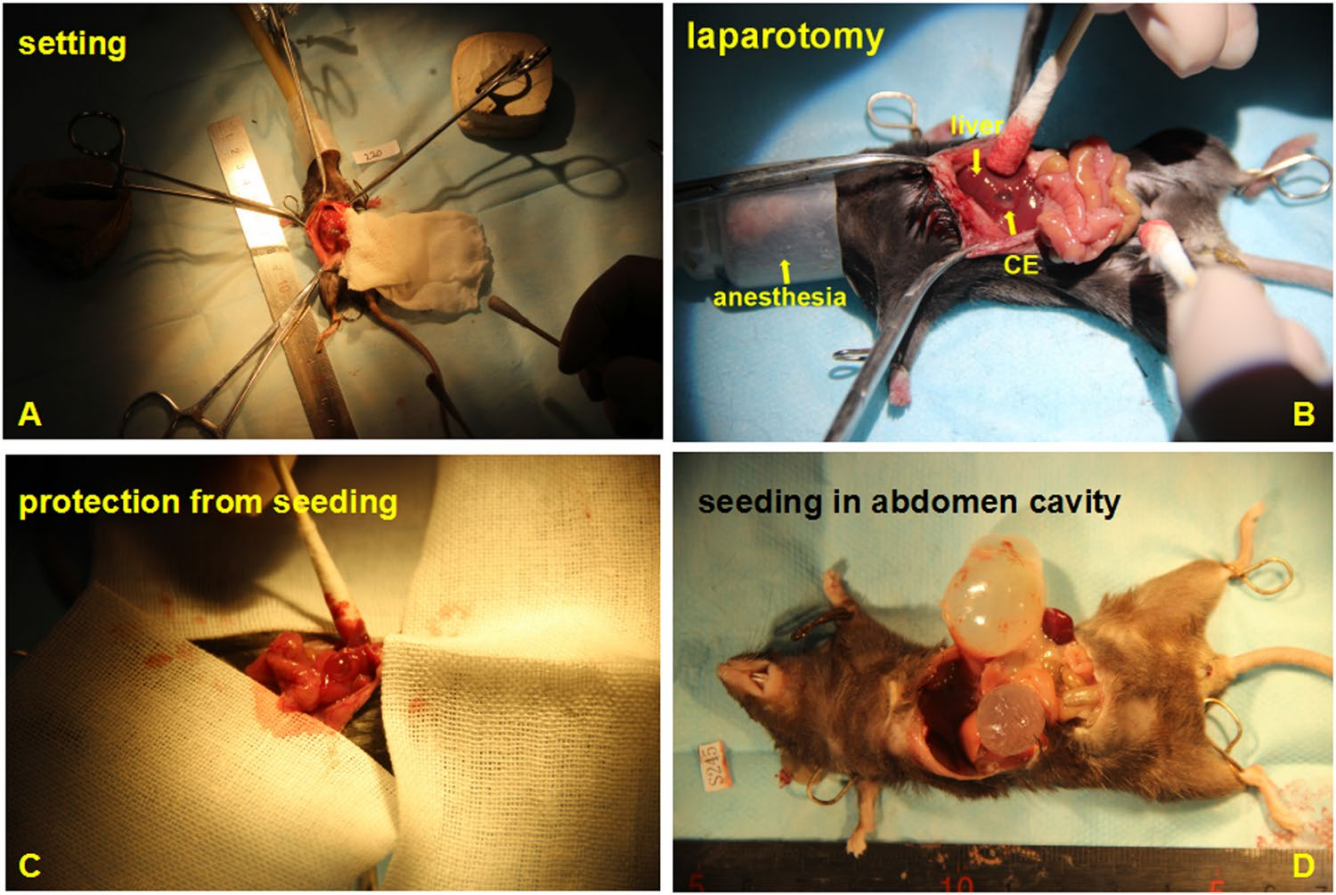
The standard therapeutic procedure surgery. (1) surgery setting; (**B**) the procedure; (**C**) special protection to prevent parasite planting; (**D**)The post surgery complication of parasite seeding in the abdomen cavity.

During nsPEF ablation, the electrodes were placed on the lateral wall of the hydatid cyst wall and achieved therapeutic effect. This placement can avoid puncture into the cyst and prevent cyst fluid spillage. One week after nsPEF, there was temporary WBC increase in nsPEF treated group but it was recoverable after 8 weeks. There is no anaphylactic reaction nor mortality during nsPEF and surgery. There was no bleeding or other post treatment complication found during follow up. Even the preventative scolicidal cheomotheray of albendazole had been routinely given and careful protection taken during surgery (Fig. 8), the post-surgery parasite planting still occurred as well as other complications:. residual cavity abscesses, 3 cases (25%); wound infection, 2 cases (17%); hydatid planting in abdomen 1 case (8%); Total 6 cases of complications (50%). The common post-surgery complications in human such as hypernatremia, deep venous thrombosis, empyema, diaphragmatic injury were not found in this murine model series.

## Discussion

Epidemiological survey showed that the prevalence of hydatid disease in China was 3.1% ~ 31.5%, seriously affecting human health and economic development. Surgery is still the most important treatment of liver cystic hydatid disease. But the recurrence and complications after surgery make the clinical efficacy is still not satisfactory^8^. Some surgical resection cannot achieve radical purpose and generally patients need to undergo multiple operations. Routine surgery cannot solve the problems caused by the violations of large blood vessels for fear that uncontrollable intraoperative bleeding. Large blood vessels, bile duct and nerve are made of collagen fibers and connective tissue. Their electrical conductivities are different from hepatocytes, so theoretically hydatid disease is indicated for nsPEF ablation. But how the structure of hydatid cyst reacts to nsPEF is unknown. It is necessary to investigate the pulse parameters in the *in vivo* application with the reference of the minimally invasive Interventional procedures successfully applied benign liver hydatid cysts, e.g. PAIR, three-dimensional conformal radiotherapy, radiofrequency ablation, high-intensity focused ultrasound^9^.

Our data showed that the anti-hydatid effect is associated with nsPEF dose. Only high electric field strength (21 kV/cm) has the significant inhabitation effect on the parasite cyst growth. There is also the limit for the parasite cyst size. Small cyst with diameter less than or equal to 1 cm was chosen to be treated by nsPEF, comparable to the human tumor size of ≤ 3 cm can be completely ablated by radiofrequency ablation^10^.

The study also proved the advantages of nsPEF ablation: it can maintain the integrity of hydatid cyst but destruct the inner layers, remain the normal surrounding liver tissue to the maximum extent; NsPEF can also be repeated and monitored by ultrasound.

Compared to conventional electrical pulses in micrometer scale, nanosecond pulsed electric field technology compress higher energy (kilovolt levels) in shorter pulses (nanoseconds). An ultra-short pulse duration, coupled with more rapid rising time, can lead to unique biological effect. E.g., 21 kV/cm for nanosecond pulses in this study compared to 1 kV/cm for conventional micrometer pulse electroporation. Previous studies assumed that nsPEF can affect the structure of cellular membrane to form tiny “nanopores”^11^. While our study use a cyst model other than cellular model to study the biological effect in an macro model with measurable and visible changes.

With the change of the electric field parameters, the mechanism of the biological effects produced by different pulse widths, different field strengths, different frequencies and different pulse numbers is different. In this experiment, some electric parameters were fixed (50 pulses, pulse duration 300 ns and frequency 1 Hz). The field strength was changed from 0, 7, 14 to 21 kV/cm. NsPEF applied on hydatid cyst has triggered a variety of effects; the parasite cyst growth initiation was due to the direct destructive effects of the electric field on parasite structure. As a new physical ablation method, nsPEF has been proved safe and effective in mouse hepatic hydatid cyst. But the further big animal trials are still needed to get the comparable dose in human.

Here we present a novel percutaneous treatment hydatid cyst with non-thermal puled electric field ablation, providing a possible alternative to open surgery with minimally invasive procedure of fewer post-surgery complications. 1. NsPEF showed inhibitory effect on liver cystic echinococcus *in vivo* on a murine hepatic hydatid cyst CE1 type. 2. NsPEF caused the direct destructive damage on the cyst wall. When the electric field strength is high enough (21 kV/cm), it can inhibit parasite growth significantly. It can also stimulate the host immune reaction by forming the inflammatory response zone and clean the debris. But the current dosage can not break the pre-existing immune tolerance formed in the chronic parasite infection. 3. The electrodes were placed on the lateral wall of the hydatid cyst wall and achieved therapeutic effect. This placement can avoid puncture into the cyst and prevent cyst fluid spillage. Although it has been used in tumor ablation and approve effective in tumor eradication and metastasis inhibition, it is still relatively new in hydatid cyst and requires much more experimental trials especially in big animals before being clinically used.

## Material and Method

### Animal experiment ethic statement

Altogether 72 female 8-weeks old C57B/6 mice were purchased from Animal Center of Xinjiang Medical University and then maintained in the Animal Laboratory of the First Affiliated Hospital of Xinjiang Medical University (License number: SCXK 2010-0003). The animal experimental proposal was approved by the Institutional Animal Care and Use Committee of the First Affiliated Hospital of Xinjiang Medical University (IACUC-20141217003). All methods were performed in accordance with the relevant guidelines and regulations of The Declaration of Helsinki and National Institutes of Health Guide for Care and Use of Laboratory Animals.

### *Echinococcus granulosus* model set up in C57B/6 mouse liver

Altogether 72 C57B/6 mice were included in this animal experiment. Among 60 mice, 12 were kept as normal without any *echinococcus granulosus* injection. The other 60 mice were injected with *echinococcus granulosus* in mouse liver. After injection of 0.003 mg atropine in muscle, the C57B/6 mouse was put on mask for continuous inhalation anesthesia. Fixed on the operating table, 1.5 cm incision was made on the middle mouse abdomen. The intestine was removed to the left side of the abdominal cavity with sterile wet saline gauze to reveal the portal vein. The sterile syringe which was pre-filled with 200 *echinococcus granulosus* protoscolices in 100ul saline was injected in the portal vein. After 28 weeks, ultrasonography detected the cystic echinococcosis grew up in liver and the diameters were about 1 cm. IgG is a marker that reflects the host-parasite immune reaction. When a hydatid cyst develops in the liver, host IgG in the serum was significantly elevated, indicating an active parasite infection. So in this study hydatid activity has been confirmed by murine serum IgG. Then the mice with active cystic echinococcosis infection in liver were treated by nsPEF.

### The classification of cystic echinococcosis in mouse liver

In 2003 the World Health Organization Informal Working Group on Echinococcosis (WHO-IWGE) published standardized classification CE1-CE5 based on ultrasound images in order to promote the stage-specific management. In our current study, CE1 type, the single complete cystic echinococcosis cysts were induced and chosen for the nsPEF treatment. Because CE1 type is the most active among all types. It facilitates a more rational treatment approach evaluation and comparison.

### Experiment design and grouping

Altogether 72 mice were divided into 5 groups. 12 mice without *echinococcus granulosus* were kept as normal for the immune baseline reference. The 60 mice with *echinococcus granulosus* liver were sent for nsPEF treatment. They were randomly divided into 5 groups with 12 mice in each group: control mice without nsPEF but received the same anesthesia and electrode placement; surgery group with standard open surgical procedure; nsPEF groups with three different dosages (Fig. 1).

### Nanosecond pulse ablation

The nsPEF generatorand electrode (Fig. 2) were applied on C57/B6 mice with parasite cyst in liver in a supine position with feet fixed onto the operating table. The pair electrodes were put on the two sides of the echinococcosis cyst. The electrode tips were punctured into the surrounding liver tissue without poking into the parasite cyst (Fig. 2).

### Surgery indication and procedure

Because the following characters in this murine series meet surgery indication: cyst locate superficially and easy to rupture; with daughter cysts and hydatid sand; communicate with biliary vessels. So the standard therapeutic procedure, laparotomy, was performed. The purpose of surgery is to completely remove the cysts and their contents. According to the WHO guideline, Four weeks before surgery and 4 weeks after the surgery the scolicidal chemotherapeutic albendazole (0.2 mg/mouse/day, intragastric administration) was given to kill the residue parasite and prevent the secondary infection. When the abdomen was opened, the surrounding tissue was covered by the gauze soaked with 20% hypertonic saline to protect from parasite planting caused by accidental spillage of the cystic contents. After full exposure of liver and lesion. The hydatid cyst was evacuated by gentle suction. The daughter cyst and hydatid sand were completely removed. The cyst-biliary tract communication was identified and repaired. The cyst cavity was sterilized with 20% saline. Then the abdomen was rinsed and closed (Fig. 8).

### Post nsPEF treatment follow-up

After nsPEF treatment, the mice were recovered in the warm pad and then kept in the animal facility with free access to water and food supplies. Before treatment, the 1st, 4th, 8th week post nsPEF, the mice were routinely checked by ultrasound, tail vein blood collection and liver biopsy. At the 12nd week post nsPEF, the mice were euthanized by CO_2_ inhalation. Blood and liver sample were collected for liver function test and pathological exams.

### Blood sample collection

During the 1st–8th post-nsPEF treatment follow-up period, the blood sample was taken from tail vein. Wwhite blood cell count was tested by an automated hematology count analyzer (Siemens, Erlangen, Germany). Liver function and cytokine were tested by automatic biochemical analyzer (Olympus AU2700, Tokyo, Japan). IgG was determined by nephelometric technique (Beckman Array 360; Beckman Coulter Instruments, Brea, U.S.A). The percentages of lymphocytes CD4/CD8 were detected by Flow-cytometry by IgG1-FITC, monoclonal fluorescent antibodies, CD4-FITC/CD8-FITC (Beckman Coulter, USA).

### Liver pathology

On euthanasia, the mouse liver tissues were sectioned into 3 mm slices and then fixed in 4% formalin for 48 hours at 4 °C. Then the tissues were dehydrated through a series of graded ethanol baths to displace the water, and then infiltrated with wax. The wax blocks were sectioned into 5 μm slides and stained by H&E. The results were reviewed in the Pathological Department of the First Affiliated Hospital of Xinjiang Medical University.

### Morphology characteristics monitored by ultrasound

A portable ultrasound system (GE Logiq e ultrasound machine, scan head 3.5 MHz, GE Health-care China Co., Ltd. Shanghai, China) was used to monitor the hepatic hydatid cyst. The diameter of the hydatid cyst, the growth was measured over time. The images were reviewed based on the standardized classification of ultrasound images of hydatid cyst by the World Health Organization Informal Working Group on Echinococcosis (WHO-IWGE) published in 2003.

### Statistical analysis

The measurement results were present as the mean ± SEM and were analyzed by one-way ANOVA followed by the Tukey’s test and a t-test. The statistical analysis was compared between groups by a software (SPSSV17.0, Chicago, IL, U.S.A). P values less than 0.05 were considered significant.
